# Structural heterogeneity of the μ-opioid receptor’s conformational ensemble in the apo state

**DOI:** 10.1038/srep45761

**Published:** 2017-04-03

**Authors:** Diniz M. Sena, Xiaojing Cong, Alejandro Giorgetti, Achim Kless, Paolo Carloni

**Affiliations:** 1 Laboratory of Computational Biophysics, German Research School for Simulation Sciences GmbH, Joint venture of RWTH Aachen University and Forschungszentrum Jülich, 52425 Jülich, Germany; 2 Computational Biomedicine section, Institute of Advanced Simulation (IAS-5), Institute of Neuroscience and Medicine (INM-9), Forschungszentrum Jülich, 52425 Jülich, Germany; 3 Departamento de Química Biológica, Universidade Regional do Cariri, Av Cel Antonio Luis 1161, 63100-000, Crato, Brazil; 4 Department of Biotechnology, University of Verona, Ca’ Vignal 1, Strada Le Grazie 15, I-37134 Verona, Italy; 5 Grünenthal Innovation, Grünenthal GmbH, 52078 Aachen, Germany

## Abstract

G-protein coupled receptors (GPCRs) are the largest and most pharmaceutically relevant family of membrane proteins. Here, fully unbiased, enhanced sampling simulations of a constitutively active mutant (CAM) of a class A GPCR, the μ-opioid receptor (μOR), demonstrates repeated transitions between the inactive (IS) and active-like (AS-L) states. The interconversion features typical activation/inactivation patterns involving established conformational rearrangements of conserved residues. By contrast, wild-type μOR remains in IS during the same course of simulation, consistent with the low basal activity of the protein. The simulations point to an important role of residue W293^6.48^ at the “toggle switch” in the mutation-induced constitutive activation. Such role has been already observed for other CAMs of class A GPCRs. We also find a significantly populated intermediate state, rather similar to IS. Based on the remarkable accord between simulations and experiments, we suggest here that this state, which has escaped so far experimental characterization, might constitute an early step in the activation process of the apo μOR CAM.

G-protein coupled receptors (GPCRs) are the largest protein family of transmembrane receptors in eukaryotes with ~800 members in the human genome^1^. They are responsible for ~80% of cell trafficking^2^, constituting the targets of almost half of FDA-approved drugs^3^. Agonist binding (photon absorption in the case of the photoreceptor rhodopsin) steers conformational and functional changes, leading to the activation of its cognate G-proteins in the cytoplasm. This in turn triggers downstream signaling events^4^. Most insights into GPCR activation have emerged for class A (or rhodopsin-like) receptors^4^, accounting for ~85% members of the family^1^: the activation involves specific conformational changes in the seven transmembrane helices (TM1–7), especially in highly conserved motifs (called at times ‘intramolecular switches’, Fig. 1). Namely, the intracellular end of TM6 bends by as much as ~10 Å away from the helical core^5^,^6^, whereas that of TM7 moves toward the core^7^. The latter, along with the intracellular halves of TMs 2, 3, and 6 rearrange so as to open a ‘hydrophobic barrier’^4^. This concerts with the so-called ‘rotamer toggle switch’ to form a water channel connecting the extra- and intracellular sides^8^,^9^,^10^,^11^. Between the toggle switch and the hydrophobic barrier lies the allosteric sodium binding pocket (Fig. 1), which collapses upon activation. Indeed, sodium binding may stabilize inactive states (IS) while being incompatible with active states (AS)^12^.

Constitutive activity has been observed for many wild type (WT) and mutated GPCRs in the *apo* state^13^,^14^, including the human μOR^15^,^16^. In constitutively active mutants (CAMs), ligand-independent activity of the receptor is notably higher than the WT, while agonist-induced activity may or may not be affected^16^,^17^. While possible roles of known mutations from the sequence/structural aspects have been discussed^18^,^19^, molecular simulations of CAMs have provided valuable insights into GPCRs activation^13^,^14^,^17^,^20^,^21^,^22^ and on the function of other membrane proteins^23^,^24^,^25^. However, little is known about the impact of CAM on GPCR’s entire conformational ensemble. Hence, a comprehensive molecular description of GPCR constitutive activation remains obscure. This important issue is investigated here, for the first time, using *apo* μOR as a test case. Specifically, we use molecular dynamics simulations to study the activation of the *apo* μOR N150^3.35^ A CAM (superscript refers to the Ballesteros-Weinstein numbering^26^). This is a particularly effective CAM as it is more active than WT μOR in the presence or absence of agonists^16^. The markedly different activity between the WT and the CAM is believed to arise, at least in part, from the disruption of the allosteric Na^+^ binding site, present across class A GPCRs^12^. Indeed, the ion stabilizes IS, and reducing Na^+^-binding may facilitate the transition towards activation^12^.

We performed replica exchange with solute scaling (REST2)^27^ molecular dynamics (MD) simulations of the CAM and of the WT *apo* μOR. With this enhanced sampling method, each system underwent 20 ns × 64 replicas MD at different temperatures, for a total of 1.28 μs. The initial structure was based on the X-ray crystal structure of μOR IS covalently bound with an irreversible antagonist, β-funaltrexamine (β-FNA) (PDB ID: 4DKL)^28^. We chose this crystal structure as it shows none of the well-known typical activation traits (Fig. 1)^4^. Comparison between the CAM and WT *apo* μOR shows that the CAM converts frequently from IS to AS-like and intermediate states. One of the states shares activation features with the AS. By contrast, the WT remains trapped in the IS.

## Results

Transitions from GPCR inactive to active states are rare events estimated to take place at millisecond timescale^29^. Hence, here we do not use straightforward MD, which typically covers a much shorter (microsecond) timescale than that of the transitions (apart from notable exceptions^30^,^31^). The REST2^27^ scheme used here is one of the so-called “enhanced sampling methods”. It enhances free energy barrier crossing, allowing sampling of rare events with much shorter computational efforts. The 1.28 μs of REST2 simulations collected here do not correspond to actual dynamics but rather to the sum of multiple discontinuous dynamics. Only the replica of the original canonical ensemble (the one at room-temperature) is analyzed and presented below. The other high-temperature replicas serve solely to enhance the sampling within the REST2 scheme. Thus, the trajectory obtained from the REST2 MD does not correspond to a progressive pathway of single conformations, but rather to interconversions among ensembles of conformations.

**Figure 1:**
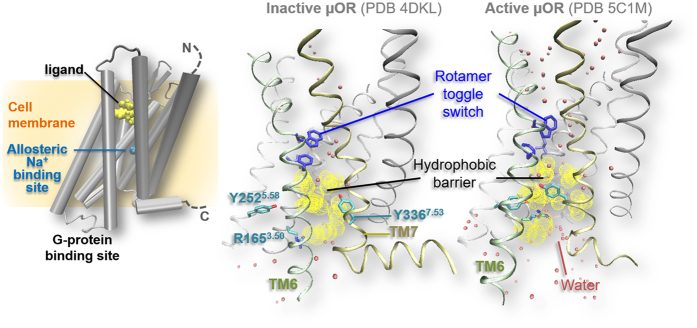
Class A GPCR structure (left) and activation patterns (right) represented here by μOR X-ray structures.

**Figure 2:**
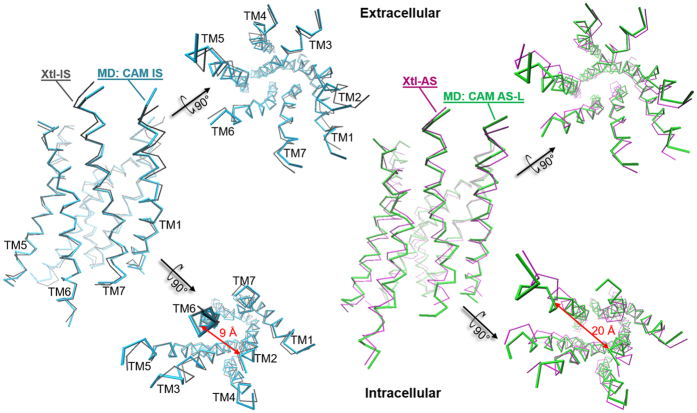
Simulated CAM IS (blue) and AS-L (green) structures superimposed onto the correspondent X-ray structures of the WT protein (gray and magenta). Only the TM helices are shown (Cα’s RMSD 0.9 Å and 1.8 Å, respectively). The distance between I279^6.34^ and T103^2.39^ Cα atoms (double-headed red arrow) increases from ~9 Å (IS) to 15 ± 0.9 Å (AS-L), indicating the dramatic outward displacement of TM6 upon activation, typical of Class A GPCRs.

Comparison of the simulated ensemble with X-ray crystal structures of μOR IS (Xtl-IS) and AS (PDB ID: 5C1M, “Xtl-AS” hereafter)^8^ shows that the CAM never reaches the fully active state as that in the available agonist- and nanobody-bound Xtl-AS^8^. Indeed, solution-state NMR has shown that both the agonist and the G protein mimetic nanobody are required to stabilize full AS of μOR^10^. However, one of the states does share most of the known activation features (Fig. 1) with Xtl-AS. We call this state an “active-like” state (AS-L, hereafter). During the simulation, the CAM interconverts many times between IS (95% overall population of the predicted ensemble) and AS-L (5% population, Fig. 2) states, suggesting the adequate sampling of the states. Hence, our simulations lead us to suggest that, in the *apo* receptor, the fully active state as obtained by agonist and nanobody binding in Xtl-AS is absent. The WT remains trapped in IS and compares fairly well with the CAM IS, except for minor differences due to the mutation (SI-1).

Cluster analysis of the simulated ensemble (see SI-Methods) shows that the CAM AS-L consists of only one cluster. The latter overlaps fairly well with Xtl-AS (Fig. 2, Movie 1). The intracellular end of TM6 in the CAM AS-L is displaced outward from the helical bundle (Fig. 2). This is the most characteristic structural feature of class A GPCR AS^5^,^6^. However, in the absence of G protein the TM6 outward displacement in the CAM AS-L is less pronounced as that in Xtl-AS (Fig. S2). A similar feature is reported for active-intermediate-like GPCR crystal structures without G protein, including the NTS1 mutants in complex with neurotensin (PDB IDs: 5T04^17^ and 4XEE/4XES^32^) and the adenosine A2A receptor in complex with adenosine (PDB ID: 2YDO^33^).

**Figure 3:**
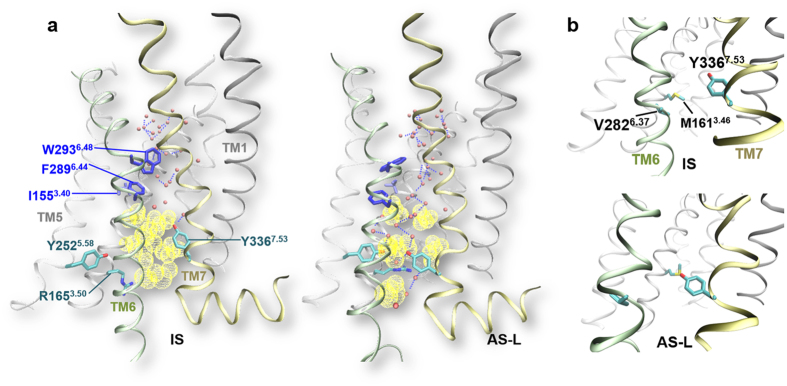
Typical activation features arising from CAM IS to AS-L transitions. (**a**) The toggle switch (blue) rotation concerts with the opening of the hydrophobic barrier (yellow dots) and formation of the water channel upon activation. Water molecules inside the channel are shown in red spheres. Blue dashed lines indicate hydrogen bonds. (**b**) Rearrangements of V282^6.37^, M161^3.46^ and Y336^7.53^ upon activation: Y336^7.53^ interacts with M161^3.46^ and V282^6.37^ is released. This latter interaction is monitored here by the minimal sidechain distance between M161^3.46^ and V282^6.37^ (Fig. S4b).

The IS↔AS-L transitions reproduce several of the common structural features (Fig. 1) of agonist-induced conformational changes in class A GPCRs (Fig. 3, Movie 2)^4^: at the rotamer toggle switch (F289^6.44^/W293^6.48^/I155^3.40^/P244^5.50^), I155^3.40^ rotates and locks between W293^6.48^ and F289^6.44^ (Fig. 3a and Fig. S3a). TM5 at the P244^5.50^ induced bulge moves toward F289^6.44^ (Fig. S3b). The hydrophobic barrier below it (residues I107^2.43^, L110^2.46^, L158^3.43^, M161^3.46^, M281^6.36^, V282^6.37^ and V285^6.40^) opens to form a water channel (Fig. 3a and Fig. S4a). V282^6.37^, M161^3.46^ and Y336^7.53^ rearrange to release V282^6.37^ from the core (Fig. 3b and Fig. S4b), which is important for G protein activation as recently proposed^34^. R165^3.50^ in the highly conserved “DRY motif”^35^ extends into the water channel to the position for binding G protein (Fig. 3a and Fig. S5a), as seen in crystal structures of GPCR AS bound to a G protein^36^. The intracellular half of TM7 comprising the N^7.49^P^7.50^x^7.51^x^7.52^Y^7.53^ motif moves inward and the allosteric Na^+^-binding site collapses. Moreover, Y336^7.53^ moves to the center and reaches Y252^5.58^ to extend the hydrogen-bond network from the water channel toward TM5 (Fig. 3b and Fig. S4b). The above features take place concurrently (Figs S2–S5 and Fig. 5). However, the TM6 outward displacement is slightly more pronounced in part of the CAM AS-L cluster (2% out of the 5% population), as reflected in Figs S2a and S4a.

The rest of the CAM structural ensemble consists instead of five other major microstates (Cα’s RMSD <1.5 Å with respect to Xtl-IS, Fig. S6), as shown by principal component analysis (PCA)-based clustering. Four of them constitute the IS (I-IV, Cα’s RMSD 1.2 ± 0.1 Å–1.4 ± 0.1 Å with respect to Xtl-IS, Fig. S1a), and the other one is likely an intermediate state (INT, Cα’s RMSD 1.4 ± 0.2 Å).

**Figure 4:**
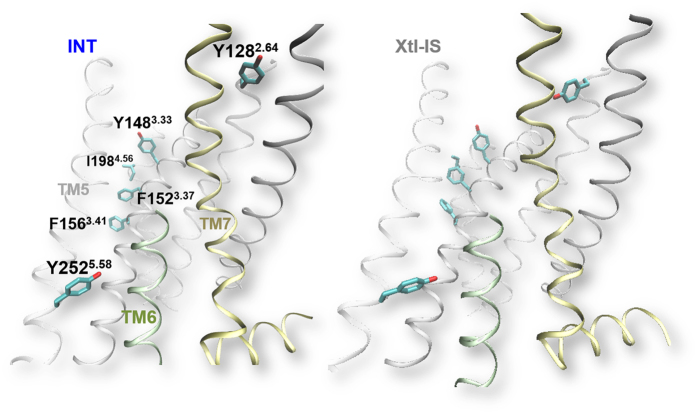
The predicted INT structure from enhanced sampling simulations of *apo* N150^3.35^A μOR. Here, the side chains of Y128^3.33^, Y148^3.33^, F152^3.37^, F156^3.41^, I198^4.56^ and Y252^5.58^ display concerted reorientation when compared with Xtl-IS (right). In the WT ensemble, only F152^3.37^ and I198^4.56^ reorient in one out of four clusters (26% population). For clarity, the extracellular half of TM6 is not shown.

The INT consists of 28% of the ensemble (Fig. S6). We suggest that this is an intermediate state because it exhibits “Xtl-AS-like” features at the orthosteric pocket. In particular, Y128^2.64^ inserts between TM1 and TM7, and the orthosteric pocket shows a general twist, similarly to what happens in Xtl-AS (Fig. S7). However, these features are not accompanied by typical GPCR activation traits shown in Fig. 1. We have compared INT with the above-mentioned active-intermediate-like states of GPCRs X-ray structures^17^,^32^,^33^. INT does not show features typical of these active states. Rather, it shows unique features that are not seen in experimentally characterized microstates so far. Specifically, residues Y128^2.64^, Y148^3.33^, F152^3.37^, F156^3.41^, I198^4.56^ and Y252^5.58^ reorient simultaneously (Fig. 4), and the orthosteric binding site shape changes (Fig. S8). In particular, Y252^5.58^ moves to an opposite direction to that in AS-L, as can be observed by measuring its distance to Y336^7.53^ (Fig. 4 and Fig. S5b). We conclude that INT is an intermediate state not yet observed in GPCR structures.

## Discussion

The four CAM IS states comprise about two thirds of the whole ensemble (populations in Fig. S6). They are quite similar to the Xtl-IS, slightly differing only, as expected, at the mutation and the orthosteric site. In particular, TM6 is located exactly as in the Xtl-IS (Fig. 2) and the typical GPCR’s activation traits (Fig. 1) are absent.

The interconversion between inactive and active states observed for the CAM, but not for the WT, are consistent with the relatively high and low basal activities of *apo* N150^3.35^A μOR and *apo* WT μOR, respectively^16^. The CAM AS-L differs from the Xtl-AS mainly in the orientation of Q124^2.60^, Y128^2.64^ and W293^6.48^ at the orthosteric site and in the position of TM6 and TM7 (Fig. S9). These are likely due to the absence of the co-crystallized ligand and the nanobody in Xtl-AS^8^, respectively. Despite the relatively large RMSD from the Xtl-AS, CAM AS-L reproduces mostly the well-known structural features of GPCR active state (Figs S2–S5 and Table S1). Hence, CAM features a transition from IS to a partially active state. By contrast, the WT shows none of these features under the same enhanced-sampling simulation conditions. Therefore, it is clearly the presence of the mutation that promotes the transition to AS. This finding must be considered as a true prediction, as our simulations do not have any prior information either on AS or on other intermediate microstates between IS and AS.

To examine whether the WT and the CAM show similar conformational changes, we analyzed the dot product of the first 9 principal components. These account for more than 65% of the variance (Fig. S12 a and b). It turns out that the CAM and the WT show rather different principal components (Table S3). This is not unexpected as the CAM undergoes transitions to the intermediate state and the active-like state, whereas the WT mainly fluctuates near the initial inactive state (Figs S14 and S15).

Which structural aspects of the CAM contribute to facilitating the transitions to AS? In an effort at addressing this issue, we have compared structural differences between the CAM IS and the WT (Table 1). A direct impact of the mutation in the CAM is the elimination of one of the allosteric Na^+^-binding residues. Consequently, Na^+^ ion binding lifetime (cumulative residence time fraction) at this site is largely reduced in the CAM with respect to that in the WT (Table 1). This is consistent with the notion that Na^+^ ion stabilizes IS. Hence, reducing Na^+^ binding may facilitate activation^12^.

**Figure 5:**
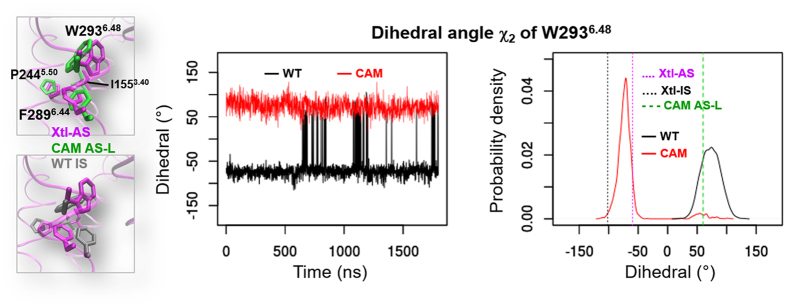
Sidechain dihedral angle χ_2_ of W293^6.48^ in the CAM (red lines) and the WT (black lines) trajectories. Left panels plot the measures in the CAM (red lines) and the WT (black lines) trajectories. Right panels show the probability density of the dihedrals, in which dashed vertical lines indicate the corresponding values in Xtl-AS (magenta), Xtl-IS (black) and CAM AS-L (mean value of the cluster, green).

A second difference is given by the conformation of one residue, W293^6.48^, in the “toggle switch”, near the mutation site. This residue plays a major role in activation by changing its conformation^4^. Our simulations indicate that this is the case in the CAM and it is not in the WT (Fig. 5 and Table 1). The change of conformation of W293^6.48^ is in line with MD studies on GPCR CAMs other than the one studied here^17^,^20^. In particular, these studies have shown that W293^6.48^ orientation differs substantially on passing from CAM to non-CAM mutants and the WT^17^,^20^. The same is true here. We conclude that the CAM may facilitate activation by changing conformation of W293^6.48^.

The upper half of TM3 (near the mutation site) and the lower half of TM7 are more flexible in the CAM than the WT (Fig. S11a). This emerged from a “core”-residue analysis (see “Methods”). An intramolecular community network analysis (see “Methods”) shows that these two regions in the CAM are less coupled to the neighboring regions than those in the WT (Fig. S11b). These may contribute to facilitating the transition of the CAM from IS to AS-L, as also suggested by Krumm *et al*. about the NTS1 CAM^17^.

INT shows similar conformation at the orthosteric pocket to that in Xtl-AS despite the absence of ligand. In particular, residue Y128^2.64^, conserved across opioid receptors, has been suggested to play a role in ligand binding and μOR conformational changes^37^. In CAM INT, this residue inserts between TM1 and TM7, and the orthosteric pocket resembles that in Xtl-AS. Being confident about the predicting power of our calculations (that reproduce the known structural features and traits of activation), here we suggest that INT might represent a very early intermediate state that escaped so far experimental characterization. Such intermediate state is possibly highly unstable in WT and thus difficult to capture experimentally. This could be a reason why this state is not seen neither in X-ray nor in NMR structures of apo GPCRs.

Can these results provide insights into agonist-induced activation in the receptor? We suggest that such interpretation should be made with caution. There exists evidence that constitutively active WT μOR activates individual G-proteins differently than an agonist^15^, and agonist-induced conformational changes in the neurotensin receptor 1 differ from those in a CAM^17^. Here we have demonstrated the power of the enhanced sampling approach– the so called REST2. This approach can be readily applied to study agonist-induced activation. Work is in progress in our lab to address this fascinating issue.

The N150^3.35^A mutation in *apo* μOR diminishes Na^+^ binding at the allosteric site and the inhibitory effect of the ion on activation. The mutation impacts on the neighboring toggle switch conformation, particularly on W293^6.48^. This in turn may trigger activation independent of agonist, as suggested for the NTS1 CAM^17^. The overall receptor structure is more flexible with less intramolecular coupling than in the WT. The mutation also induces a novel state with Xtl-AS-like features at the orthosteric site, which may represent an intermediate state that favors agonist binding. This state may also reduce the energy barrier of activation, thus facilitating this process.

## Methods

The initial models of μOR WT and CAM were both based on the inactive X-ray crystal structure of μOR (PDB code: 4DKL)^28^. The WT model was built using the procedure described in our previous work (see SI-Method)^38^. The N150^3.35^A mutation was introduced using the Swiss PDB Viewer^39^. Hydrophilic cavities in the models were detected and pre-filled with water using the DOWSER program^40^. The g_membed tool^41^ was used to embed each protein model in a bilayer of 1-Palmitoyl-2-oleoyl-sn-glycero-3-phosphocholine (POPC), the most abundant phospholipid in animal cell membranes^42^. The system was then solvated in a periodic 67 × 70 × 107 Å^3^ box of explicit water and neutralized with 0.15 M NaCl. Finally, the simulation system consisted of ~49,000 atoms, including ~9,450 water molecules, 28 Na^+^ and 41 Cl^−^ ions.

The “Stockholm Lipids”^43^, Amber99SB-ildn^44^, TIP3P^45^ and the Joung-Cheatham^46^ force fields were used for the lipids, the protein, the water molecules and the ions, respectively. The simulation system was energy minimized and gradually heated to 300 K. REST2 simulations were then performed in the *NPT*-ensemble (*P* = 1 bar, *T* = 300 K) with 64 replicas, applying the Andersen-Parrinello-Rahman barostat^47^,^48^ and the Nose-Hoover thermostat^49^. The effective temperature ranged from 300 K to 550 K, following a distribution calculated with the Patriksson-van der Spoel approach^50^. This choice led to an exchange probability ranging from 34% to 63% (~55% on average). All the simulations were carried out with Gromacs 4.6^51^. The CAM and the WT each underwent 20 ns × 64 replicas of MD simulations. Discarding the first 2 ns, trajectories at 300 K were analyzed. Residues experiencing the least fluctuations (the “core” residues) were identified using the Bio3d program and 1 Å^3^ volume cutoff^52^. Intramolecular community network analysis was performed using the NetworkView plugin for VMD^53^. PCA was employed to identify and characterize clusters of structures, just as is usually done in protein folding dynamics^54^. These were carried out with Gromacs tools^51^. More details of the simulation and analyses can be found in SI-Methods.

## Additional Information

**How to cite this article:** Sena Jr., D. M. *et al*. Structural heterogeneity of the µ-opioid receptor’s conformational ensemble in the apo state. *Sci. Rep.* **7**, 45761; doi: 10.1038/srep45761 (2017).

**Publisher's note:** Springer Nature remains neutral with regard to jurisdictional claims in published maps and institutional affiliations.

## Supplementary Material

Supporting Information

Supplementary Movie 1

Supplementary Movie 2

Supplementary Movie S1

## Figures and Tables

**Table 1:** Selected differences between the CAM and the WT (mean ± standard deviation are given when applicable).

	CAM	WT
Allosteric Na^+^ ion lifetime	2%	18%
W293^6.48^ sidechain orientation (χ_2_ dihedral angle)	IS: −74.3 ± 11.4	73.2 ± 16.2
I155^3.40^ sidechain orientation (χ_1_ dihedral angle)	IS: −60.4 ± 5.8	−59.9 ± 8.0
P244^5.50^-F289^6.44^ minimal sidechain distance	IS: 5.8 ± 0.9	6.5 ± 0.4
Number of “core” residues (see “Methods”)	50	65
Intramolecular community network	CAM shows weaker coupling around the mutation site, in the upper half of TM3 and the lower half of TM7 than the WT	
