# Supplementary material for: Structural heterogeneity of the μ-opioid receptor’s conformational ensemble in the apo state
Source: Sci Rep. 2017 Apr 3;7:45761. doi: 10.1038/srep45761 (PMC5377942; doi:10.1038/srep45761)
Supplement: Supporting Information [file srep45761-s1.pdf]

Structural heterogeneity of the μ-opioid receptor's conformational ensemble in the apo state

Diniz M. Sena Jr[1,2,3,&], Xiaojing Cong[1,2,&,*], Alejandro Giorgetti[2,4], Achim Kless[5], Paolo Carloni[1,2,*]

1 Laboratory of Computational Biophysics, German Research School for Simulation Sciences GmbH, Joint venture of RWTH Aachen University and Forschungszentrum Jülich, 52425 Jülich, Germany

2 Computational Biomedicine section, Institute of Advanced Simulation (IAS-5), Institute of Neuroscience and Medicine (INM-9), Forschungszentrum Jülich, 52425 Jülich, Germany

3 Departamento de Química Biológica, Universidade Regional do Cariri, Av Cel Antonio Luis 1161, 63100-000, Crato, Brazil

4 Department of Biotechnology, University of Verona, Ca' Vignal 1, Strada Le Grazie 15, I-37134 Verona, Italy

5 Grünenthal Innovation, Grünenthal GmbH, 52078 Aachen, Germany

& These authors contributed equally to this work.

* Corresponding authors:

X. C., current address: Institute of Chemistry, University of Nice Sophia Antipolis, 06108 Nice cedex 2, France, e-mail: xiaojing.cong@unice.fr,

P. C., e-mail: p.carloni@fz-juelich.de

SI-1. Predicted WT ensemble

During the molecular dynamics simulations with enhanced sampling (REST2), WT μOR remains in IS. The structural determinants are similar to those of Xtl-IS (Movie S1), especially considering the TM6-TM2 distance (Fig. 2 in main text). The Cα's RMSD of the TM region is 1.5±0.4 Å with respect to Xtl-IS. The predicted WT IS shows notable differences from the CAM IS: TM6 and part of TM7 shift downward with respect to TM3, and the rotamer toggle switch adopts a different conformation (Fig. S1). This leads to the breaking of the $D147^{3.32}$-$Y326^{7.43}$ hydrogen bond (cumulative residence time 3%, to be compared with 64% in the CAM).

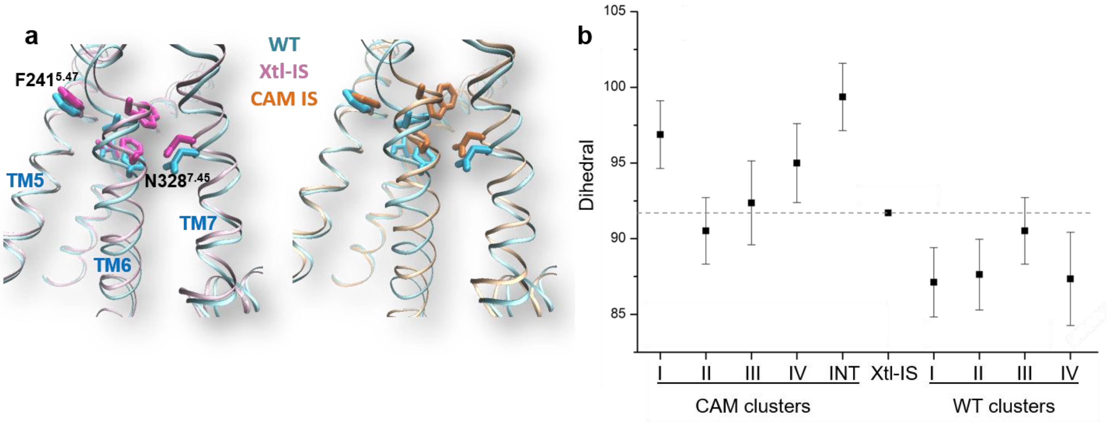

Figure S1. (a) Superimposition of a representative structure of the WT (cyan) onto Xtl-IS (magenta) and onto a representative structure of the CAM IS (orange). WT exhibits a downward shift of TM6 and altered rotamer toggle switch conformation. Part of the WT TM7 also shifts downward and outward, while $N328^{7.45}$ rotates outward. **(b)** Dihedral angle of $I107^{2.43}$-$D114^{2.50}$-$S154^{3.39}$-$F289^{6.44}$ Cα atoms indicates the downward shift of TM6 relative to TM3 in the WT clusters when compared with the CAM non-AS clusters and Xtl-IS.

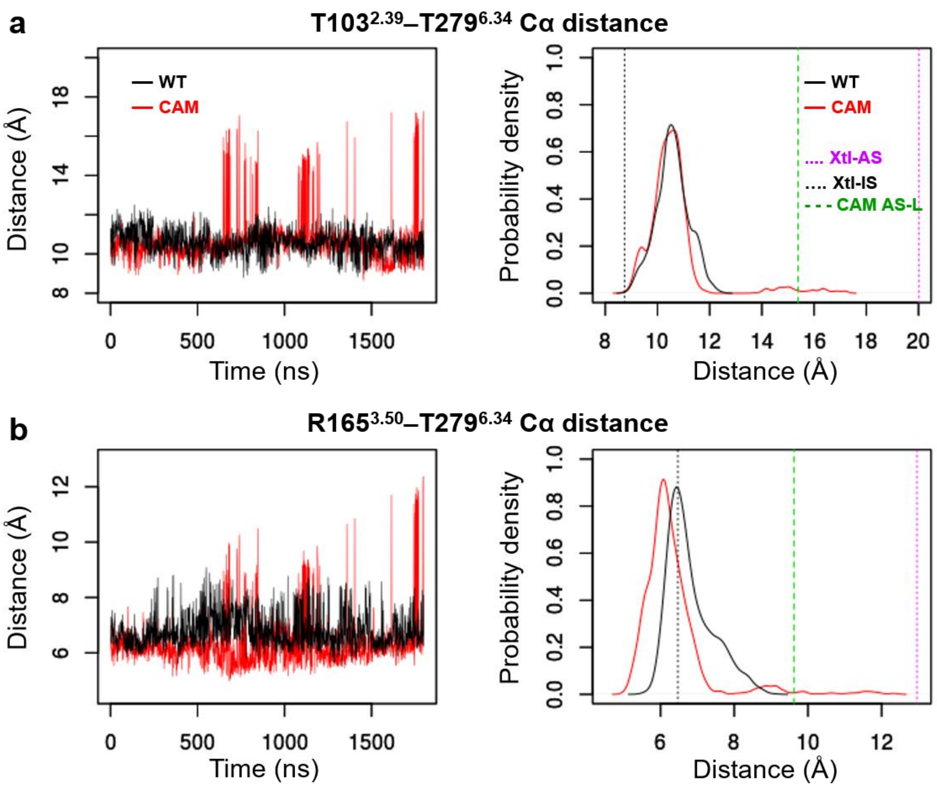

Figure S2. Distances between **(a)** T103$^{2.39}$ and T279$^{6.34}$ and **(b)** R165$^{3.50}$ and T279$^{6.34}$ Cα atoms. These are taken here as a measure of the outward displacement of the TM6 intracellular end relative to those of TM2 and TM3, respectively. The left panel reports the distances for the CAM (red lines) and the WT (black lines). The right panels show the probability density of the distances. Here, dashed vertical lines indicate the corresponding values in Xtl-AS (magenta), Xtl-IS (black) and CAM AS-L (mean value of the cluster, green). Note that here, and in figures S3–S5 and S10, simulation time is reported solely to quantify the number of steps in the REST2 simulations, without providing information on the timescale (for a discussion of this point, see Main Text).

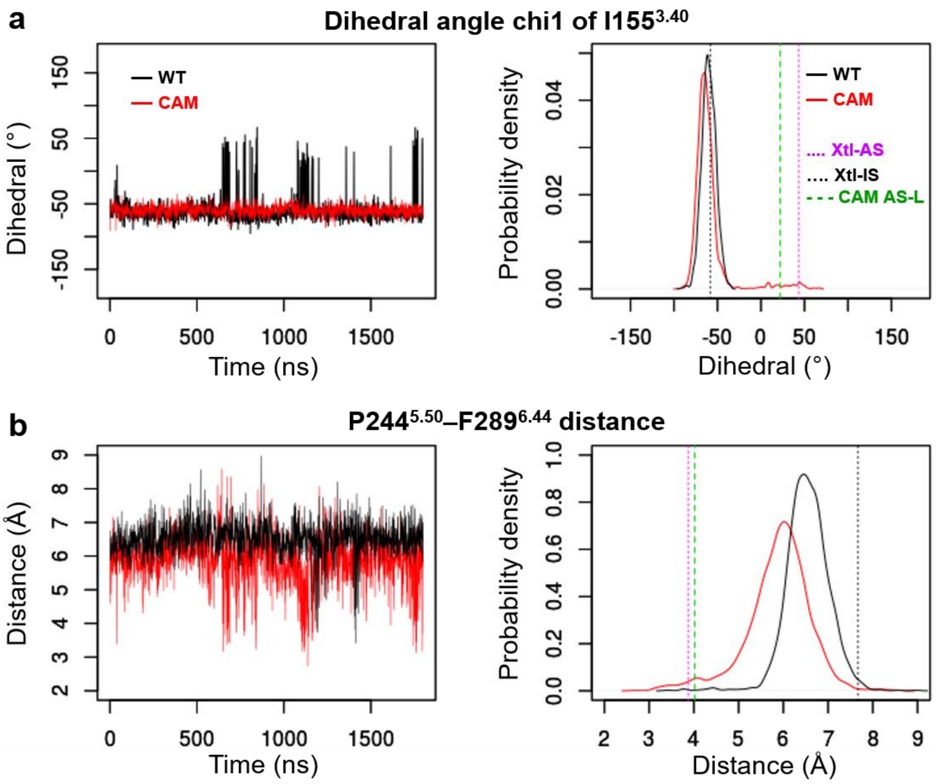

Figure S3. **(a)** Dihedral angle χ_1 of residue I155$^{3.40}$ and **(b)** minimal sidechain distance between P244$^{5.50}$ and F289$^{6.44}$ non-hydrogen atoms for the CAM (red lines) and the WT (black lines). Panel layout and color code are the same as those in Fig. S2.

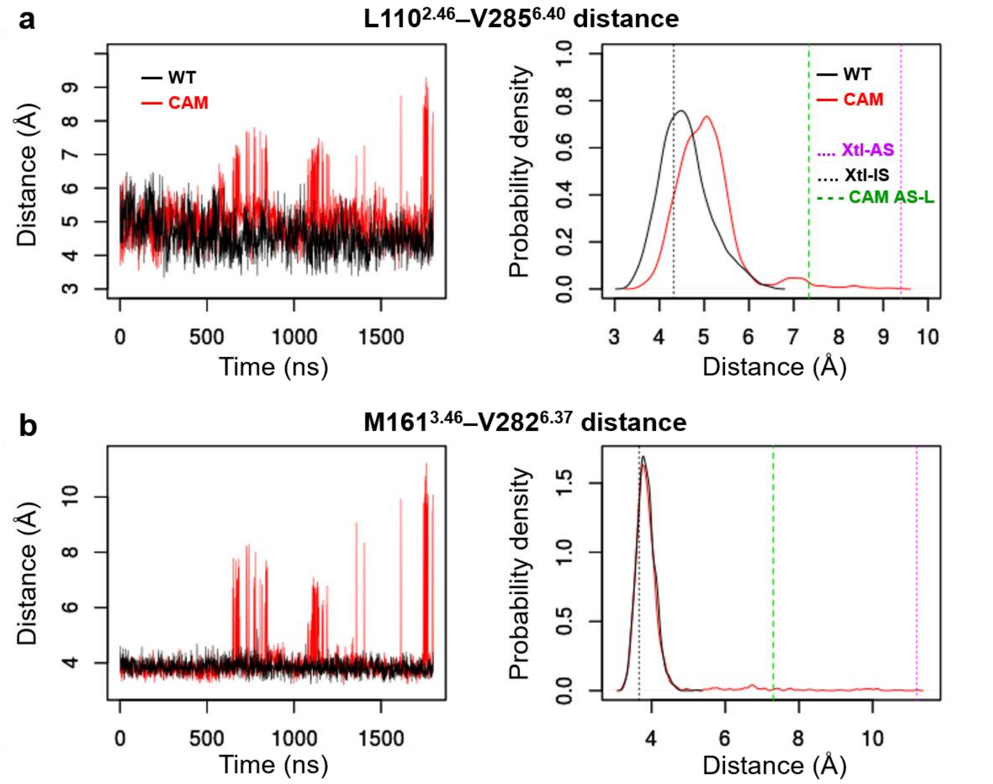

Figure S4. Sidechain distance between **(a)** L110$^{2.46}$ and V285$^{6.40}$ and **(b)** M161$^{3.46}$ and V282$^{6.37}$ in the CAM (red lines) and the WT (black lines) trajectories. The distances are taken from the closest non-hydrogen atoms of the residue pairs during the simulation. Panel layout and color code are the same as those in Fig. S2.

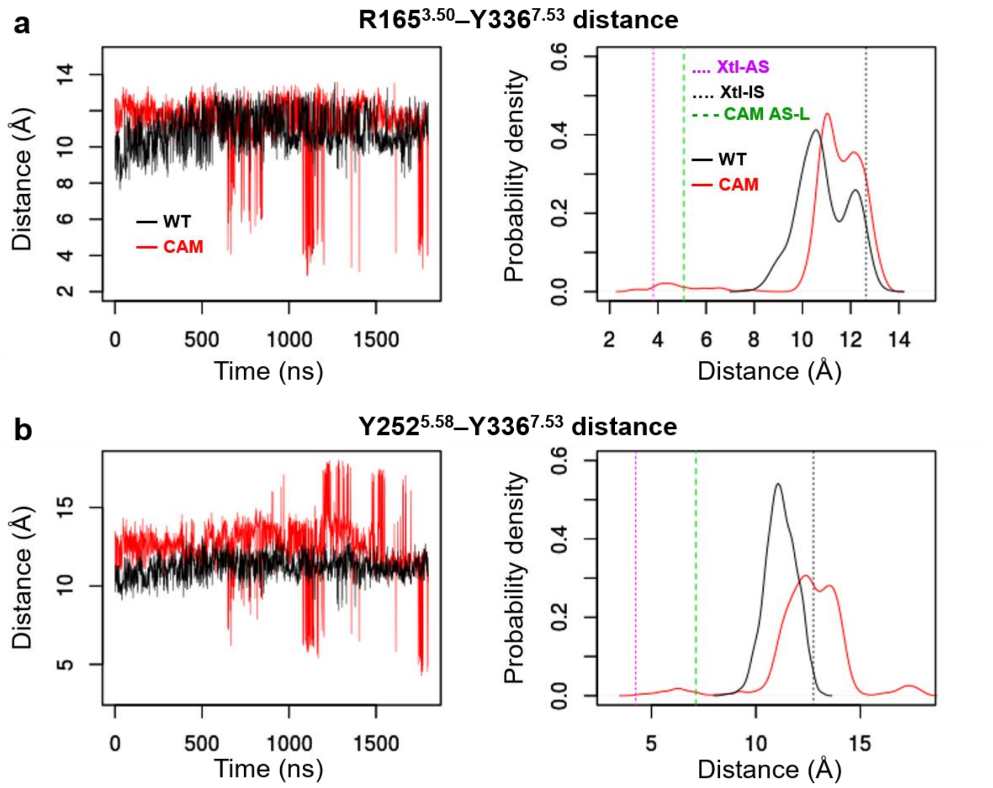

Figure S5. Minimal sidechain distance between **(a)** R165$^{3.50}$ and Y336$^{7.53}$, and **(b)** Y252$^{5.58}$ and Y336$^{7.53}$ in the CAM (red lines) and the WT (black lines) trajectories. The distances are taken from the closest non-hydrogen atoms of the residue pairs during the simulation. Panel layout and color code are the same as those in Fig. S2.

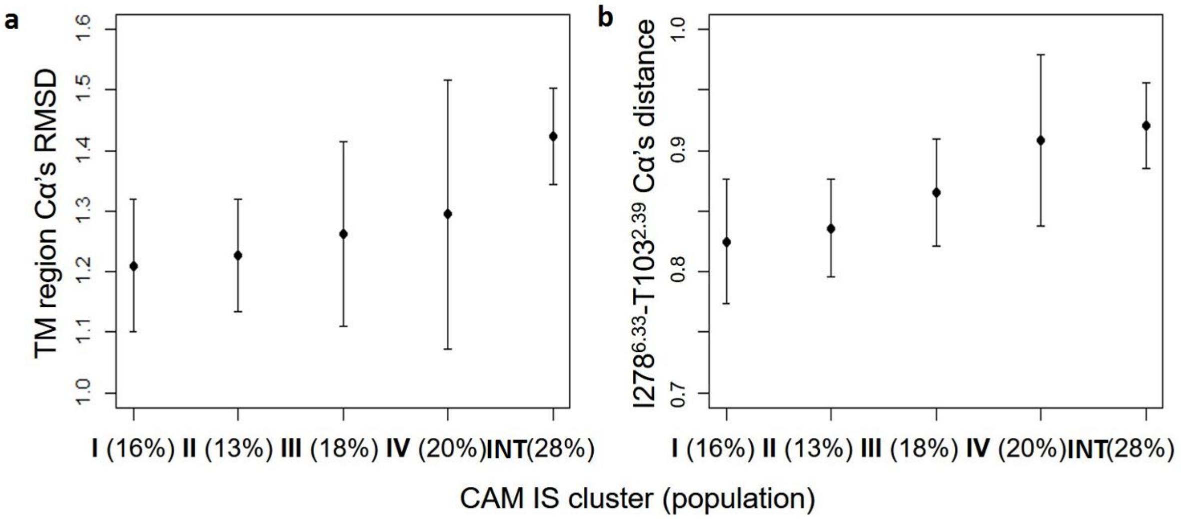

Figure S6. Microstates of the computed structural ensemble of *apo* N150$^{3.35}$A μOR other than AS-L. **(a)** Cα's RMSD of the TM region with respect to Xtl-IS. **(b)** TM6-TM2 distance of microstates **I-IV** and **INT**, which indicates no significant outward movement of TM6.

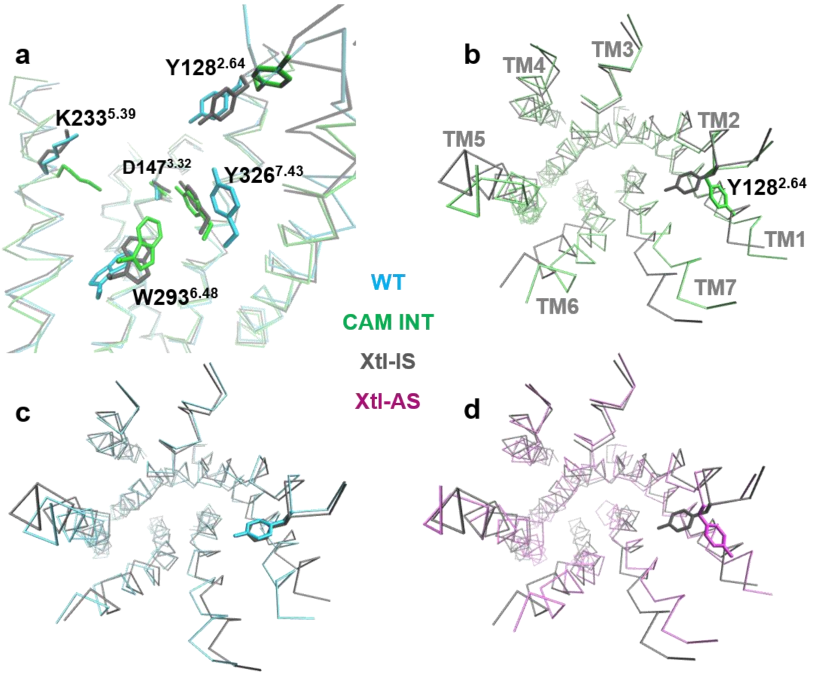

Figure S7. Orthosteric binding sites across Xtl-IS, Xtl-AS, WT and CAM INT. **(a)** WT features a W293$^{6.48}$ orientation other than Xtl-IS and a lack of D147$^{3.32}$-Y326$^{7.43}$ hydrogen bond present in CAM INT, which shows different Y128$^{2.64}$ and K233$^{5.39}$ orientations relative to Xtl-IS. Note that in Xtl-IS, the residue K233$^{5.39}$ is covalently bound to the antagonist. For clarity, TM6 and TM7 are not shown. **(b-d)** Upon the reorientation of Y128$^{2.64}$ in CAM INT, the orthosteric pocket shows an anticlockwise twist (from the extracellular view) compared to Xtl-IS and the WT. Y128$^{2.64}$ orientation and twist in CAM is similar to that of Xtl-AS, TM5 is distorted around K233$^{5.39}$ and the latter reorients into the pocket toward D147$^{3.32}$ (in **a**).

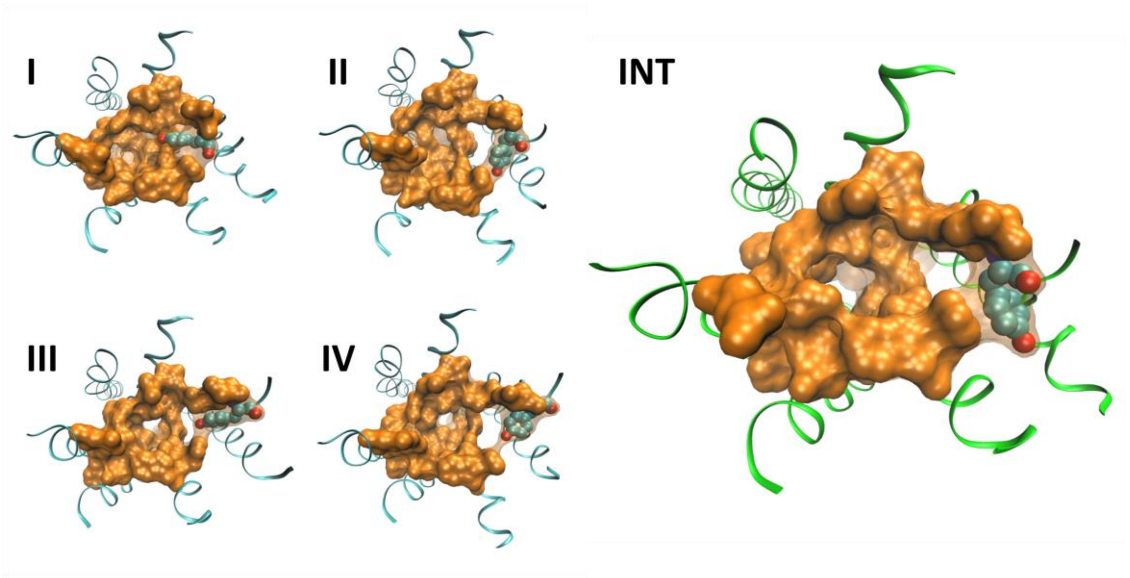

Figure S8. Changes in the orthosteric binding site shape for CAM-IS (I to IV) and CAM INT. For clarity, only TM residues are shown as ribbon, and pocket residues are shown as surface. Y128$^{2.64}$ nonhydrogen atoms are depicted as van der Waals spheres, under a transparent surface. The large movement of this residue follows the conformational change in the shape of the binding pocket.

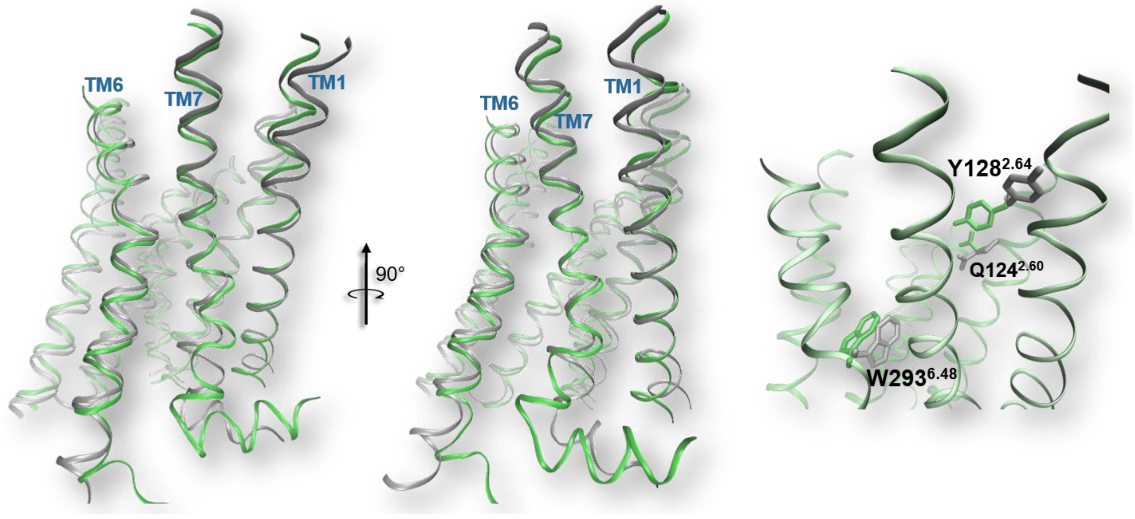

Figure S9. A representative structure of the predicted CAM AS-L (green) superimposed on Xtl-AS (gray).

Table S1. Values of the distances plotted in Figs. S2–S5 for Xtl-AS, Xtl-IS, CAM AS-L and WT. Distances are shown in Å and dihedral angles in degrees.

Residues	Xtl-AS	Xtl-IS	CAM AS-L (mean±SD)	WT (IS) (mean±SD)
T103$^{2.39}$-T279$^{6.34}$ Cα	20.0	8.7	15.4±0.9	10.6±0.6
R165$^{3.50}$-T279$^{6.34}$ Cα	13.0	6.5	9.6±1.2	6.8±0.6
P244$^{5.50}$-F289$^{6.44}$	3.9	7.7	4.0±0.5	6.5±0.4
L110$^{2.46}$-V285$^{6.40}$	9.5	4.3	7.3±0.7	4.6±0.6
M161$^{3.46}$-V282$^{6.37}$	9.6	3.7	7.3±1.6	3.9±0.2
R165$^{3.50}$-Y336$^{7.53}$	3.8	12.6	5.1±1.3	10.9±1.0
Y252$^{5.58}$-Y336$^{7.53}$	4.2	12.8	7.1±1.7	11.2±0.8
I155$^{3.40}$ dihedral χ_1	44	-58	22±28	-60±8

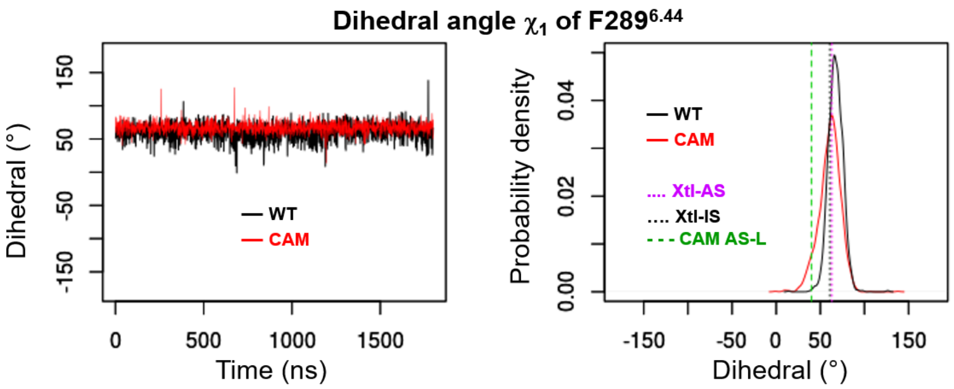

Figure S10. Sidechain dihedral angle χ_1 of residue F289$^{6.44}$ in the CAM (red lines) and the WT (black lines) simulations (Left). (Right) Probability density of the angle. Dashed vertical lines indicate the corresponding values in Xtl-AS (magenta), Xtl-IS (black) and CAM AS-L (mean value of the cluster, green).

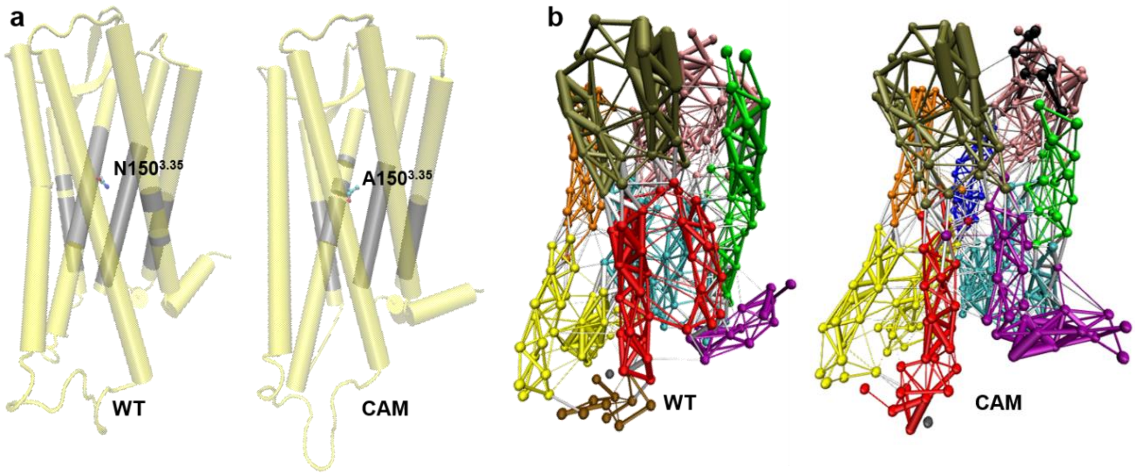

Figure S11. (**a**) Core residues (in gray, see main text Materials and methods for definition) shown in cartoon for WT and CAM. (**b**) Intramolecular interaction network of the CAM and the WT. Thicker lines indicate stronger correlation. Colors represent different communities (groups of residues with coupled motions).

SI-Methods

μOR models. The Xtl-IS structure (PDB: 4DKL) [1] contains a covalently bound antagonist (β-funaltrexamine) and a T4 lysozyme replacing the third intracellular loop (IL3). These were removed. The missing side chains and IL3 was constructed using Modeller 9.9 [2], conserving the water molecules in Xtl-IS. In total 20,000 μOR models were generated, from which the one with the lowest Discrete Optimized Protein Energy (DOPE) score [2] was selected. The NQ-Flipper [3] and H++ [4] webservers were used to examine the asparagine and glutamine side-chain rotamers and to predict the histidine side-chain protonation states, respectively.

Molecular simulations. Energy minimization was performed in 2 stages: 1) steepest-descent minimization with 1,000 $kJ \cdot mol^{-1} \cdot nm^{-2}$ harmonic positional restraints on the protein, and 2) conjugate-gradient minimization (with 1 step of steepest-descent minimization per 1000 steps) without restraints. Then, the systems were coupled to the Berendsen thermostat and barostat [5] while being heated to 300 K in 6 steps of 100-ps MD simulations (from 2 K to 60 K, 120 K, 180K, 240 K and 300 K). The velocities were generated consistent with a Maxwell-Boltzmann distribution at the corresponding temperature. REST2 simulations in the *NPT*-

ensemble used the Andersen-Parrinello-Rahman barostat [6,7] and the Nose-Hoover thermostat [8]. Semi-isotropic pressure coupling was applied throughout the MD simulations, which allows the simulation box in the *z*-axis (perpendicular to the lipid bilayer) to vary independently of the *x*-*y* plane. The LINCS algorithm [9] was employed to constrain all the bond lengths, enabling a 2-fs time step. Van der Waals and short-range electrostatic interactions were cut off at 12 Å. Long-range electrostatic interactions were computed using the Particle Mesh Ewald summation (PME) [10] method with a Fourier grid spacing of 1.2 Å.

Cluster analysis. The WT and CAM trajectories were aligned on the backbone atoms and Principal Component Analysis (PCA) was carried out on heavy atoms using gromacs tool g_covar. The cosines content of the first 9 eigenvectors are close to 0 (Table S2), indicating good sampling quality (high content close to 1 is related to random diffusion) [11,12]. The projection of vectors from WT and CAM trajectories are measured as the cosine value associated with their scalar product.

The first 16 eigenvectors account for 73% and 79% of the covariance for the WT and the CAM trajectories, respectively (Fig. S12 a and b). The bend (or elbow) in the scree plot (Fig. S12 c and d) of the sum of squared error suggest 4 clusters for the WT and 6 for the CAM. Projection of the data points onto selected principle component (PC) pairs (Fig. S13) suggest good separation of the clusters.

Table S2. Cosine contents [16] for the first 9 eigenvectors of the WT and CAM trajectories.

# eigenvectors	# periods	Cosine content	
		WT	CAM
1	0.5	2.59e-01	3.75e-02
2	1.0	1.19e-01	2.84e-03
3	1.5	9.39e-03	2.21e-02
4	2.0	1.38e-03	2.87e-05
5	2.5	7.69e-04	2.38e-05
6	3.0	1.93e-04	1.57e-03
7	3.5	8.41e-03	3.33e-03
8	4.0	2.64e-02	7.96e-03
9	4.5	2.40e-03	1.11e-02

Table S3. Superposition of eigenvectors, obtained after the scalar product between WT and CAM eigenvectors, expressed as the cosine of the angle between them.

**PCn (WT)•PCn (CAM)**	
**Eigenvector rank (n)**	cos(θ)
**1**	0.221
**2**	0.030
**3**	-0.091
**4**	0.092
**5**	0.019
**6**	-0.188
**7**	0.031
**8**	-0.041
**9**	0.028

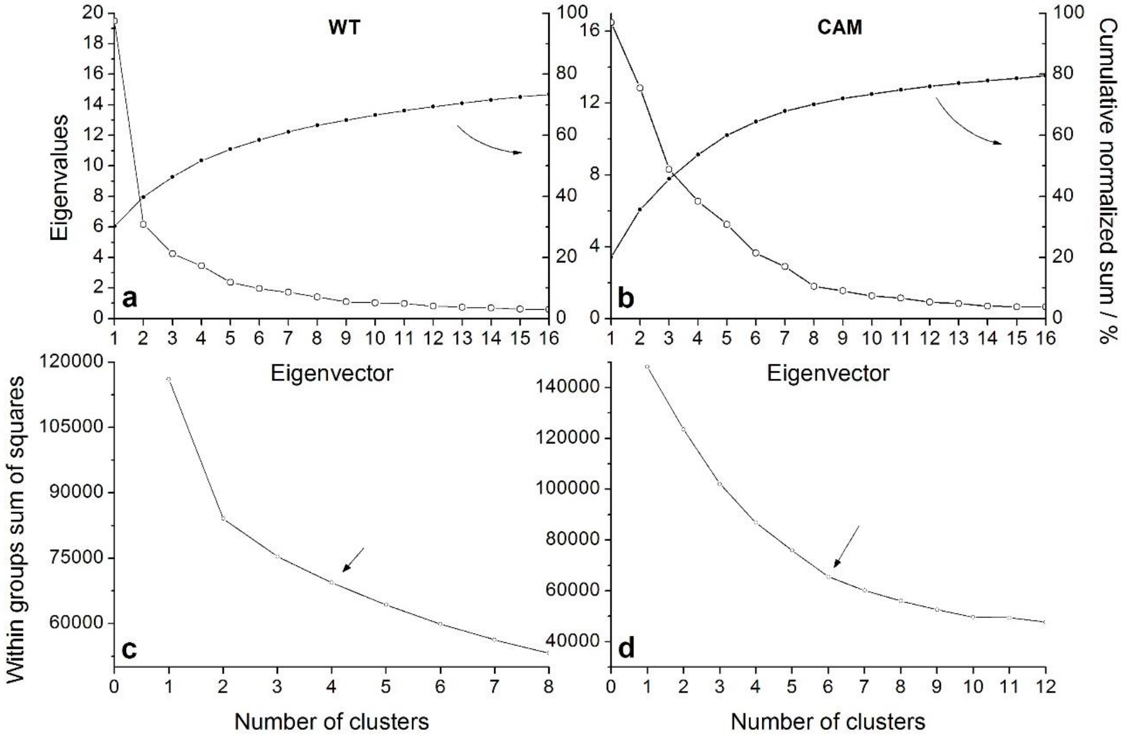

Figure S12. **(a** and **b)** Eigenvalues (open circles) and cumulative normalized sum (dots) of the first 16 eigenvectors of the WT and the CAM trajectories. **(c** and **d)** Scree plots of the sum of squared error of each point in one cluster to its centroid summed for all clusters (within groups sum of squares) [17]. Position of the elbow used to determine the optimal number of clusters is indicated.

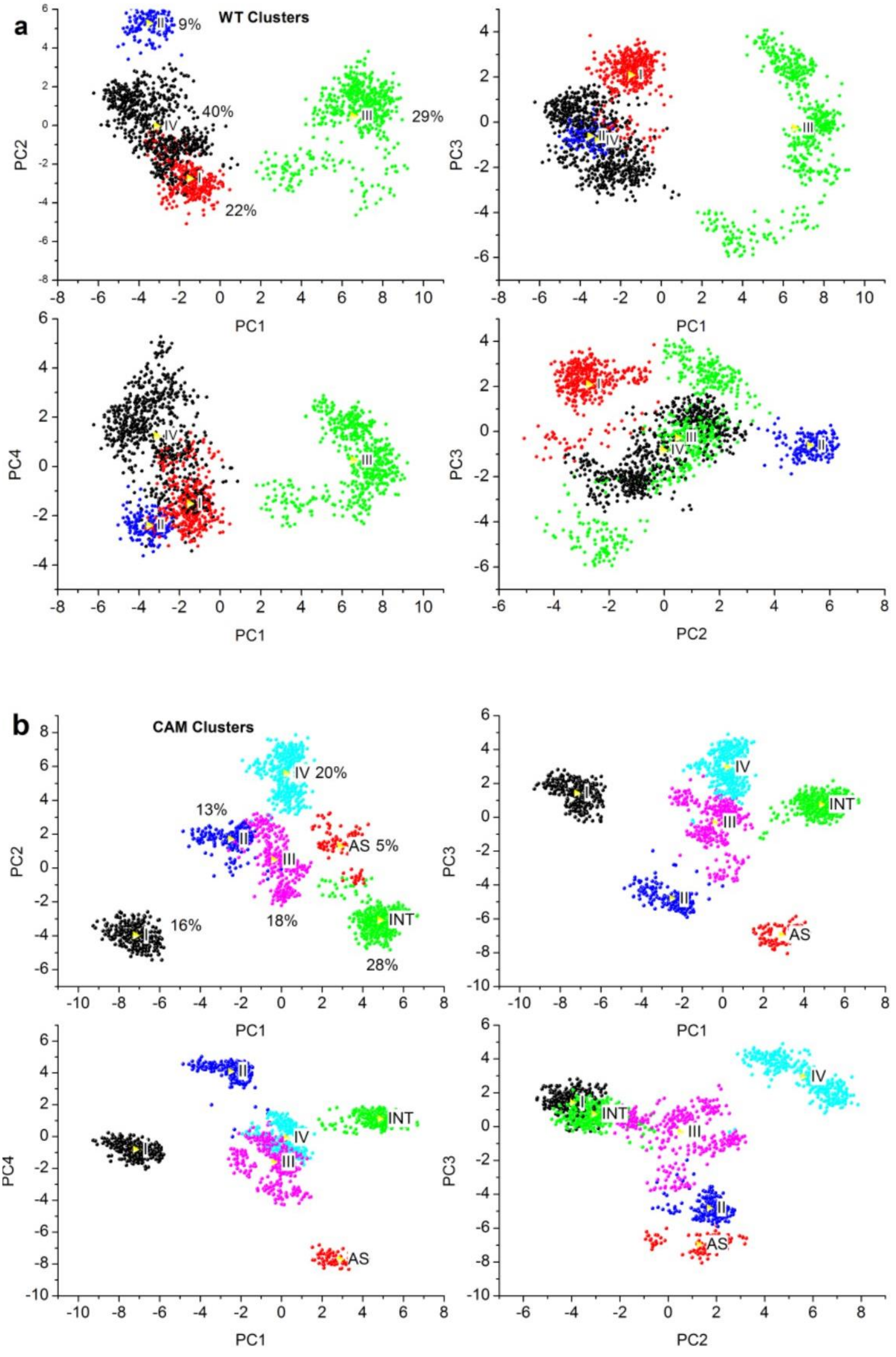

Figure S13. Projections of **(a)** the WT and **(b)** the CAM trajectories onto selected pairs of principal components (PC). Cluster numbers and their populations are indicated.

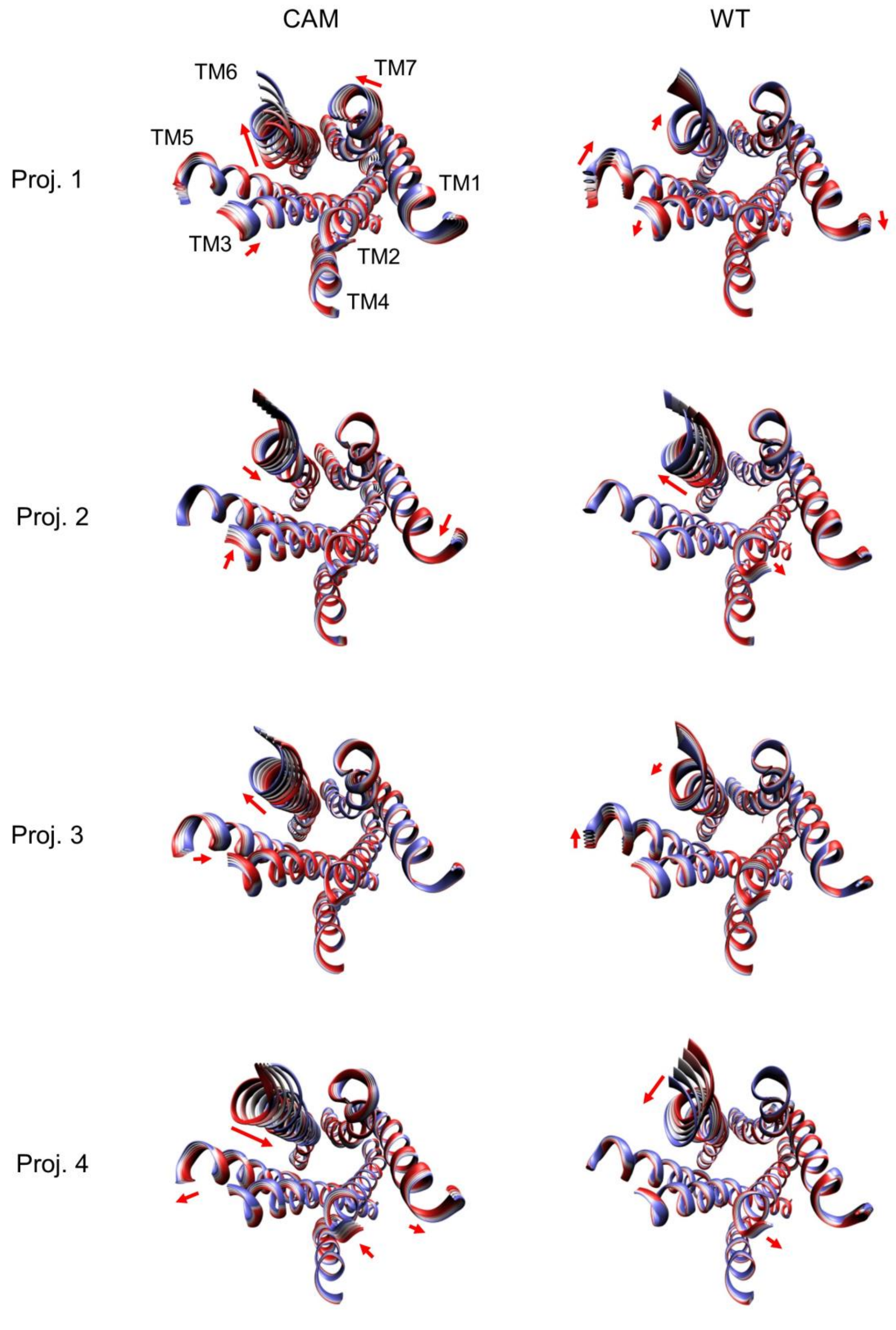

Figure S14. Intracellular view of the projections of the simulation trajectories along the first four eigenvectors on the CAM (left) and WT (right) structures. For clarity, loop residues are not shown. Transmembrane helices numbers are indicated in the first projection.

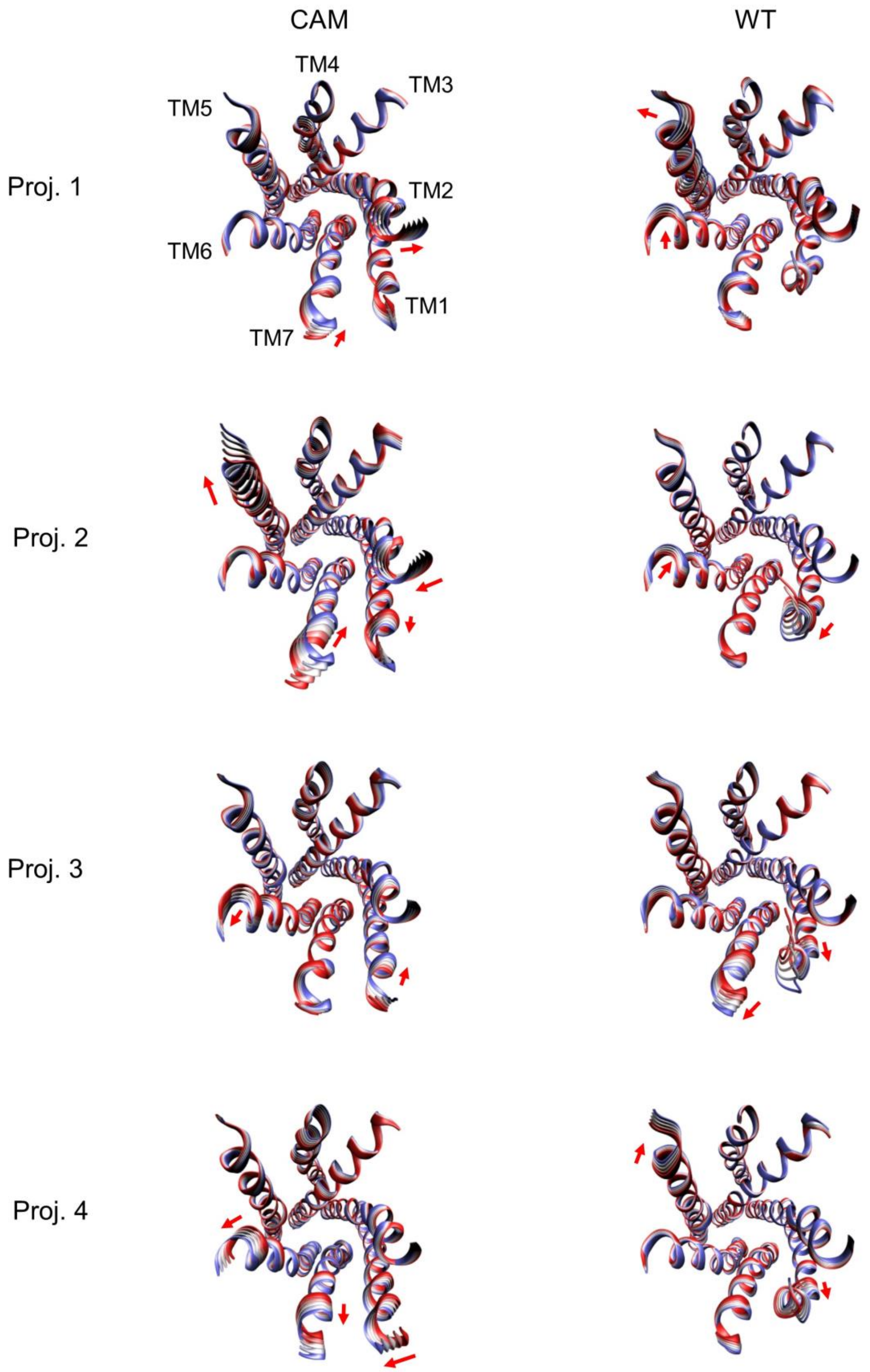

Figure S15. Extracellular view of the projections of the simulation trajectories along the first four eigenvectors on the CAM (left) and WT (right) structures. For clarity, loop residues are not shown. Transmembrane helices numbers are indicated in the first projection.

Supporting references

1 Manglik, A. *et al.* Crystal structure of the mu-opioid receptor bound to a morphinan antagonist. *Nature* **485**, 321-326, doi:10.1038/nature10954 (2012).

2 Eswar, N. *et al.* Comparative protein structure modeling using Modeller. *Curr Protoc Bioinformatics* **Chapter 5**, Unit 5 6, doi:10.1002/0471250953.bi0506s15 (2006).

3 Weichenberger, C. X. & Sippl, M. J. NQ-Flipper: recognition and correction of erroneous asparagine and glutamine side-chain rotamers in protein structures. *Nucleic Acids Research* **35**, W403-W406, doi:10.1093/nar/gkm263 (2007).

4 Gordon, J. C. *et al.* H++: a server for estimating pK(a)s and adding missing hydrogens to macromolecules. *Nucleic Acids Research* **33**, W368-W371, doi: 10.1093/nar/gki464 (2005).

5 Berendsen, H. J. C., Postma, J. P. M., Vangunsteren, W. F., Dinola, A. & Haak, J. R. Molecular-Dynamics with Coupling to an External Bath. *J Chem Phys* **81**, 3684-3690, doi:10.1063/1.448118 (1984).

6 Andersen, H. C. Molecular-Dynamics Simulations at Constant Pressure and-or Temperature. *J Chem Phys* **72**, 2384-2393, doi:10.1063/1.439486 (1980).

7 Parrinello, M. & Rahman, A. Polymorphic Transitions in Single-Crystals - a New Molecular-Dynamics Method. *J Appl Phys* **52**, 7182-7190, doi:10.1063/1.328693 (1981).

8 Nose, S. & Klein, M. L. Constant Pressure Molecular-Dynamics for Molecular-Systems. *Mol Phys* **50**, 1055-1076, doi:10.1080/00268978300102851 (1983).

9 Hess, B., Bekker, H., Berendsen, H. J. C. & Fraaije, J. G. E. M. LINCS: A linear constraint solver for molecular simulations. *J Comput Chem* **18**, 1463-1472, doi:10.1002/(Sici)1096-987x(199709)18:12<1463::Aid-Jcc4>3.0.Co;2-H (1997).

10 Darden, T., Perera, L., Li, L. P. & Pedersen, L. New tricks for modelers from the crystallography toolkit: the particle mesh Ewald algorithm and its use in nucleic acid simulations. *Struct Fold Des* **7**, R55-R60, doi:10.1016/S0969-2126(99)80033-1 (1999).

11 Maisuradze, G. G. & Leitner, D. M. Principal component analysis of fast-folding λ-repressor mutants. *Chemical Physics Letters* **421**, 5-10, doi: 10.1016/j.cplett.2006.01.044 (2006).

12 Hess, B. Convergence of sampling in protein simulations. *Physical review. E, Statistical, nonlinear, and soft matter physics* **65**, 031910, doi:10.1103/PhysRevE.65.031910 (2002).

13 Grant, B. J., Rodrigues, A. P. C., ElSawy, K. M., McCammon, J. A. & Caves, L. S. D. Bio3d: an R package for the comparative analysis of protein structures. Bioinformatics 22, 2695-2696, doi:10.1093/bioinformatics/btl461 (2006).

14 Eargle, J. & Luthey-Schulten, Z. NetworkView: 3D display and analysis of protein·RNA interaction networks. Bioinformatics 28, 3000-3001, doi:10.1093/bioinformatics/bts546 (2012).

15 Humphrey, W., Dalke, A. & Schulten, K. VMD: Visual molecular dynamics. Journal of Molecular Graphics 14, 33-38, doi:10.1016/0263-7855(96)00018-5 (1996).

16 Maisuradze, G. G., Liwo, A. & Scheraga, H. A. Principal Component Analysis for Protein Folding Dynamics. *Journal of Molecular Biology* **385**, 312-329, doi:10.1016/j.jmb.2008.10.018 (2009).

17 Wolf, A. & Kirschner, K. N. Principal component and clustering analysis on molecular dynamics data of the ribosomal L11.23S subdomain. *Journal of molecular modeling* **19**, 539-549, doi:10.1007/s00894-012-1563-4 (2013).

Movie legends

Movie 1. Superposition of CAM AS-L frames onto Xtl-AS structure.

Movie 2. Conformational changes arising from CAM IS to AS-L transitions.

Movie S1. Superposition of WT frames onto Xtl-IS structure.
